# The influence of mussel restoration on coastal carbon cycling

**DOI:** 10.1111/gcb.16287

**Published:** 2022-06-20

**Authors:** Mallory A. Sea, Jenny R. Hillman, Simon F. Thrush

**Affiliations:** ^1^ Institute of Marine Science, University of Auckland Auckland New Zealand

**Keywords:** blue carbon, benthic fluxes, benthic–pelagic coupling, bivalves, carbon, carbon budget, restoration, soft‐sediments, subtidal

## Abstract

Increasing responsiveness to anthropogenic climate change and the loss of global shellfish ecosystems has heightened interest in the carbon storage and sequestration potential of bivalve‐dominated systems. While coastal ecosystems are dynamic zones of carbon transformation and change, current uncertainties and notable heterogeneity in the benthic environment make it difficult to ascertain the climate change mitigation capacity of ongoing coastal restoration projects aimed at revitalizing benthic bivalve populations. In this study we sought to distinguish between direct and indirect effects of subtidal green‐lipped mussels (*Perna canaliculus*) on carbon cycling, and combined published literature with in‐situ experiments from restored beds to create a carbon budget for New Zealand's shellfish restoration efforts. A direct summation of biogenic calcification, community respiration, and sediment processes suggests a moderate carbon efflux (+100.1 to 179.6 g C m^−2^ year^−1^) occurs as a result of recent restoration efforts, largely reflective of the heterotrophic nature of bivalves. However, an examination of indirect effects of restoration on benthic community metabolism and sediment dynamics suggests that beds achieve greater carbon fixation rates and support enhanced carbon burial compared to nearby sediments devoid of mussels. We discuss limitations to our first‐order approximation and postulate how the significance of mussel restoration to carbon‐related outcomes likely increases over longer timescales. Coastal restoration is often conducted to support the provisioning of many ecosystem services, and we propose here that shellfish restoration not be used as a single measure to offset carbon dioxide emissions, but rather used in tandem with other initiatives to recover a bundle of valued ecosystem services.

## Graphical abstract

In this study we sought to distinguish between direct and indirect effects of green‐lipped mussels on carbon cycling, and combined published literature with in‐situ experiments from restored beds to create a carbon budget for New Zealand’s shellfish restoration efforts. A direct summation of biogenic calcification, community respiration, and sediment processes suggests a moderate carbon efflux occurs as a result of recent restoration efforts, largely reflective of the heterotrophic nature of bivalves. However, an examination of indirect effects of restoration on benthic community metabolism and sediment dynamics suggests that beds achieve greater carbon fixation rates and support enhanced carbon burial compared to nearby sediments devoid of mussels.

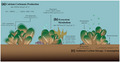

## 1 INTRODUCTION

Humankind has altered the global carbon cycle with concerning pace (Falkowski et al., 2000; Hansen et al., 2006), the physical and biological consequences of which are currently being experienced in both terrestrial and marine realms (Doney et al., 2012; Hoegh‐Guldberg & Bruno, 2010; Rosenzweig et al., 2008). Rising concerns over global environmental change have been met with a rapid increase in research outputs related to climate change mitigation and adaptation strategies (Einecker & Kirby, 2020) and the potential carbon storage/uptake capacity of natural systems, with scientific efforts largely concentrated on the ability of vegetated coastal habitats to fix and store carbon (e.g., “blue carbon” habitats such as mangroves, salt marshes, and sea grasses; Duarte et al., 2010; Mcleod et al., 2011; Alongi, 2012; Duarte et al., 2013; e.g., Macreadie et al., 2021).

Located at the land–sea interface, coastal ecosystems are dynamic zones of carbon transformation and change (Chen & Borges, 2009; Najjar et al., 2018), and their sediments have been identified as major sites of carbon burial, with coastal oceans contributing to as much as 80% of total organic carbon burial (Berner, 1982; Burdige, 2007; Keil, 2017; Rabouille et al., 2001). Storage in these sediment systems is complex and variable over space and time (Bianchi et al., 2018), involving the rate of settlement, particle mixing, and subduction of carbon deep into sediments. Large, reef‐building bivalves such as oysters and mussels are seen as important in their contributions to benthic–pelagic coupling and generating microhabitats conducive to the mediation of biogeochemical cycles (e.g., Ray et al., 2019). As carbon is also utilized in the shell formation process, there has been recent interest in understanding how globally threatened, bivalve systems influence coastal carbon cycling at ecologically relevant scales (Filgueira et al., 2015; Fodrie et al., 2017).

Conventionally, the influence of bivalves on coastal carbon cycling has been reduced to the summation of an individual's carbon dioxide (CO_2_) emissions (resulting from respiration and calcification) versus carbon stored in the individual's shell and tissue (see Filgueira et al., 2019). This, however, neglects to address the engineering role (Gutiérrez et al., 2003; Meadows et al., 2012) and upscaling effects (Sea et al., 2021) that bivalves demonstrate at the ecosystem level. For example, populations of bivalve filter feeders play a significant role in benthic–pelagic coupling (the exchange of energy and nutrients between the water column and seafloor; Griffiths et al., 2017), exerting top‐down control on carbon‐fixing phytoplankton populations and influencing nutrient availability and energy transfer in these systems (Newell, 2004). Furthermore, the physical protrusion of bivalve beds above the seafloor alters local hydrodynamics, traps sediment particles, and stabilizes the seafloor (Butman et al., 1994; Gutiérrez et al., 2003; Riisgård et al., 2011; Widdows et al., 2002), encouraging sedimentation and carbon storage in areas rich with organic material resulting from filter feeding. The effects of bivalves on carbon cycling are thus more complex than simple shell formation and dissolution equations at the individual scale, and questions regarding their implications on coastal carbon must be resolved using an ecosystem approach (e.g., Filgueira et al., 2015) which encompasses direct and indirect feedbacks (physical, biological, and biogeochemical) of bivalves on their environment. Using a more holistic standpoint, some bivalve systems have been deemed carbon sinks (e.g., Fodrie et al., 2017), although the magnitude and extent of this sink capacity across species and geographic locations is currently unknown.

There is further uncertainty surrounding the appraisal of biogenic calcification to coastal carbon budgets (Macreadie et al., 2017; Saderne et al., 2019), as this process increases *p*CO_2_ of seawater and (to a certain extent) results in atmospheric carbon emissions (Zeebe & Wolf‐Gladrow, 2001). Those focused on culturing bivalves for human consumption have suggested carbon accounting systems which split carbon fluxes attributed to shell formation from carbon fluxes associated with tissue (Filgueira et al., 2015; Filgueira et al., 2019). Others have chosen to omit shell formation from evaluations completely, acknowledging “a lack of consensus on whether calcification represents a source or sink of CO_2_” (van der Schatte Olivier et al., 2020). Uncertainties associated with shell production must be addressed if we hope to accurately assess carbon cycling in systems dominated by these calcium carbonate secreting organisms.

To understand the consequences of coastal bivalve beds in a climate context, the effects of filtration and organic matter deposition need to quantified and balanced against shell production and metabolic processes, as heterotrophic bivalves respire and produce CO_2_. When considered at the ecosystem scale, diverse benthic communities supported by biogenic reef habitat also need to be included in calculations, as they consume locally trapped organic matter before burial and respire, producing further CO_2_. Bivalve reefs will be net carbon sinks if their capacity to store organic carbon in local sediments and inorganic carbon in shell material outweighs carbon dioxide emissions resulting from ecosystem metabolism and biogenic calcification. This capacity almost certainly varies between systems and species, making it important to resolve if we are to advance shellfish restoration or conservation as a viable climate mitigation strategy.

Extensive shellfish beds with the potential to affect carbon cycling at ecologically relevant scales have largely been decimated worldwide (e.g., Airoldi & Beck, 2007; Gillies et al., 2018; Lotze et al., 2006), and restoration projects are currently underway to recover the ecosystem services they provide (Zu Ermgassen et al., 2020). With increased interest in restoring coastal habitats and mitigating anthropogenic carbon emissions, there is a significant need to determine how large‐scale bivalve restoration projects affect carbon budgets of coastal systems. In light of current uncertainties, our overall objective was to document and quantify carbon transformations following recent restoration efforts (focusing solely on carbon transformations after addition of adult mussels to soft‐sediment systems), utilizing a holistic, ecosystem‐based approach to approximate a first‐order carbon budget in restored beds. We address carbon cycling concepts by combining in‐situ and laboratory experiments with published and unpublished data obtained from subtidal mussel restoration efforts in the Hauraki Gulf of northern New Zealand, and report ranges of potential carbon uptake (−) and/or emissions (+) resulting from calcium carbonate production, ecosystem metabolism, and dynamic sediment processes. Our examination of New Zealand's restored mussel beds highlights how heterogeneity affects carbon outcomes at the bed‐scale and is important in addressing current knowledge gaps related to coastal carbon cycling and sequestration potential in restored bivalve systems.

## 2 MATERIALS AND METHODS

### 2.1 Study area and budget overview

**FIGURE 1:**
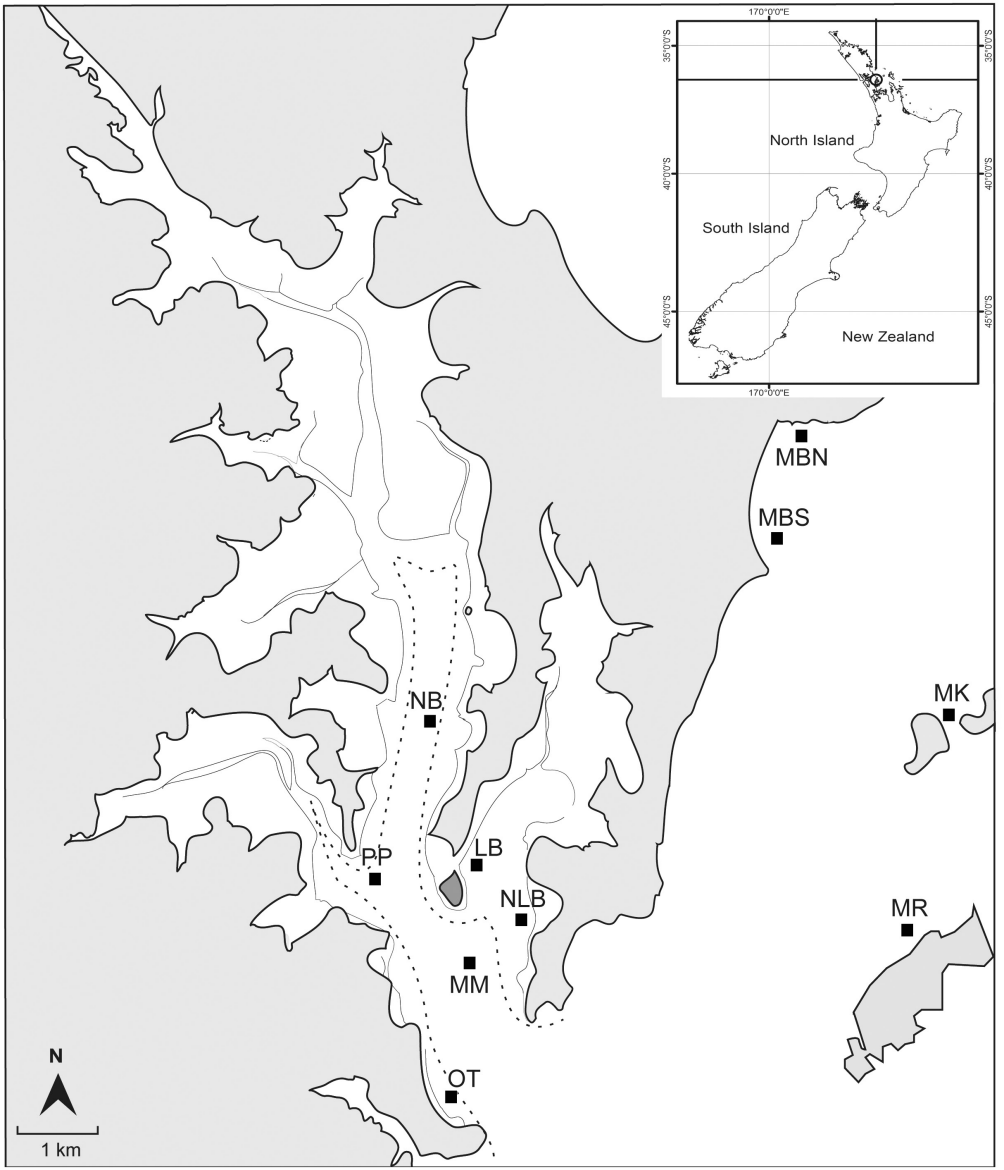
Location of mussel restoration sites in Mahurangi Harbour and Kawau Bay, New Zealand. Site labels: MM, Mahurangi Mid; MBN, Martins Bay North; MBS, Martins Bay South; MR, Motuora; MK, Motoketekete; NB, Ngaio Bay; NLB, New Lagoon Bay; LB, Lagoon Bay; OT, Otarawao Bay; PP, Pukapuka.

Where possible, our carbon budget utilizes data collected from studies of shallow, subtidal, green‐lipped mussels in the Hauraki Gulf of New Zealand. Over the past 5 years, more than 200 tonnes of adult mussels have been relocated from regional longline mussel farms to soft‐sediment locations around the Gulf, generating multiple beds near Mahurangi Harbour and Kawau Bay (Figure 1). While these mussel beds are of similar depth (5–15 m) and size (~10–25 m^2^), the locations they were restored in were chosen to encompass a range of environmental conditions (namely variations in exposure, sediment grain size, porosity, organic matter and macrofaunal assemblages) capable of affecting biogeochemical cycling at ecologically relevant scales (e.g., Hillman et al., 2021). Combining field collections with laboratory experiments and published literature, we utilized data from as many of these sites as possible (Table S1 in the supplement) to capture environmental variation and expand the generality of our results. If data necessary for the carbon budget were not available from these beds, we expanded our search to publications using green‐lipped mussels throughout New Zealand, or, if unavailable, to other bivalves in similar temperate climates.

**FIGURE 2:**
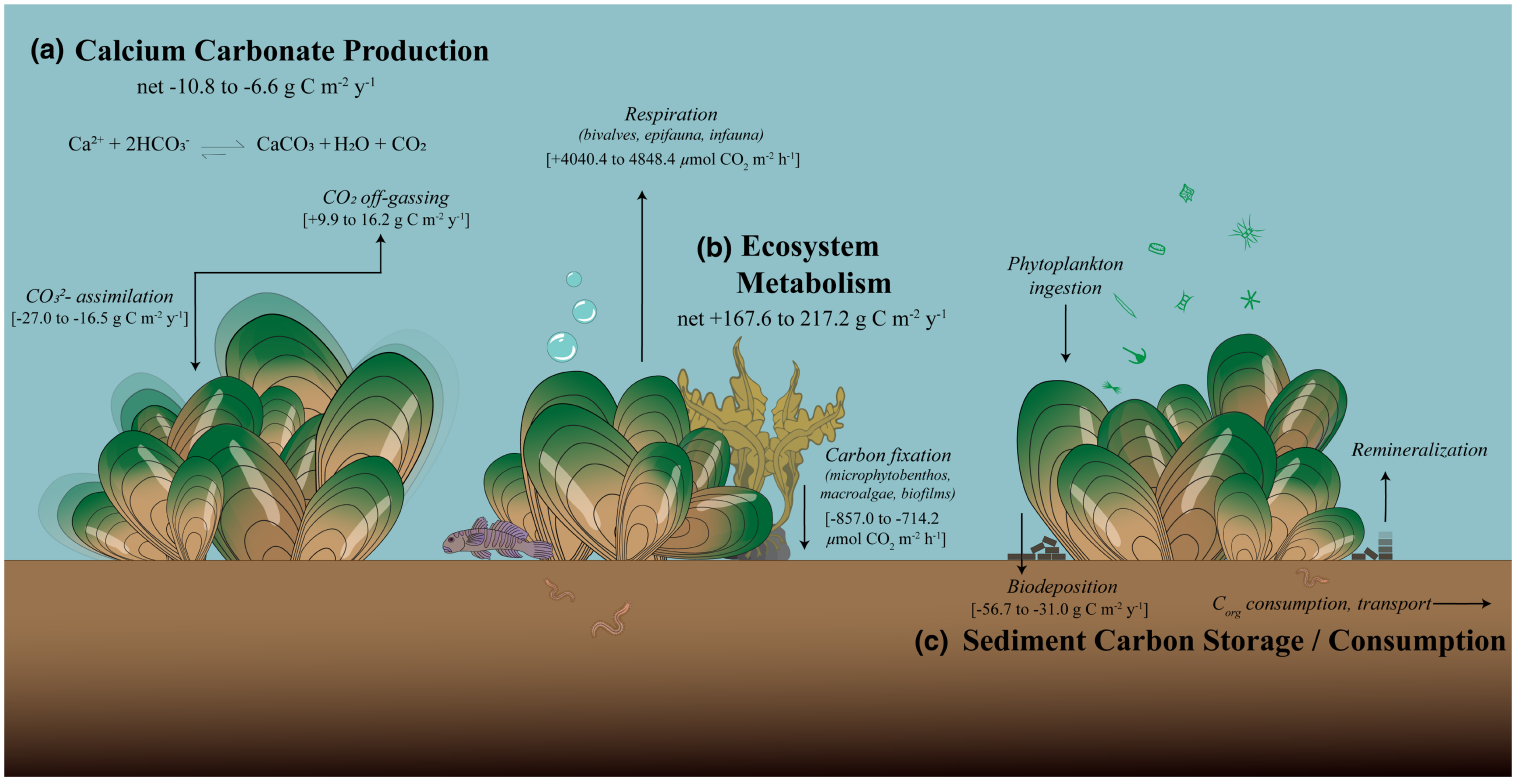
Infographic illustrating carbon cycling and transformations in restoration sites comprised of adult, green‐lipped mussels (size 95–115 mm). Negative values (−) are indicative of processes that promote carbon storage, drawdown, or fixation while positive values (+) denote carbon effluxes. Information is conceptually divided into three parts: (a) calcium carbonate production, (b) ecosystem metabolism, and (c) sediment consumption/storage. Note that part (c) has no associated total net flux, as biodeposit remineralization processes are mechanistically linked to experimental methods utilized in part (b) of the budget.

We conceptually divided the effects of mussel restoration on carbon cycling into three parts: A. mussel shell formation/dissolution; B. benthic metabolism of mussel bed communities (local respiration of the benthic communities vs. carbon fixation); and C. carbon storage in sediments beneath mussel beds (Figure 2). All calculations are based on an idealized 15 adult (95 mm‐115 mm) mussels per m^2^, an estimate designed to express the patchy spatial aggregation patterns observed on these beds (Sea et al., 2021). The following sections summarize what is known about the role of green‐lipped mussels in carbon cycling to justify selected values and calculations used to create our budget.

### 2.2 Shell formation and dissolution

Bivalves utilize bicarbonate ions (HCO_3_^−^) in seawater to synthesize a calcium carbonate shell (CaCO_3_), a process which is generally summarized using the below Equation (1):

(1) $${\mathrm{Ca}}^{2+}+2\mathrm{HC}O_{3}^{-}⇌\mathrm{CaC}O_{3}+H_{2}O+CO_{2}$$

This equation for the precipitation of calcium carbonate, however, is a rather “simplistic representation” which can lead to the mistaken conclusion that 1 mol of carbon dioxide is generated per mol of calcium carbonate produced; in reality, roughly 0.6 mol CO_2_ is produced for every mol of precipitated CaCO_3_ (Ware et al., 1992).

This effect can be attributed to the chemical nature of carbon dioxide, which dissociates in seawater and takes on four different inorganic forms which readily switch between one another and collectively regulate seawater pH (referred to as the bicarbonate buffer system or the carbonate system; Zeebe & Wolf‐Gladrow, 2001). A portion of CO_2_ produced from biogenic calcification forms HCO_3_^−^ while the remaining CO_2_ is either released to the atmosphere or inhibits additional carbon uptake by the oceans (Ray et al., 2018). While the amount of CO_2_ emitted as a result of shell formation has been shown to vary with temperature, salinity, and local carbonate chemistry (Morris & Humphreys, 2019), it is generally accepted that ~0.6 mol CO_2_ is produced for every mol of precipitated CaCO_3_ (Macreadie et al., 2017; Saderne et al., 2019). Here, we credit carbon temporarily stored in mussel shell over an average individual's lifetime, or roughly 30 years (a timescale comparable to the rotation of timber stands recognized as carbon stores in the terrestrial realm; Brockerhoff et al., 2008; Zhang et al., 2012; Xie et al., 2020). To quantify carbon stored in shell, we used the following equation (Equation 2):

(2) $$C_{s}=W*C_{f}$$

Where C_s_ is the amount of carbon stored (g m^−2^ year^−1^), W is the annual change in dry shell weight, and C_f_ is the carbon fraction of mussel shell's dry weight. Using molar ratios outlined by Ware et al. (1992), we then multiplied calculated C_s_ values by 0.6 to estimate CO_2_ production resulting from the precipitation of calcium carbonate. Other studies (Filgueira et al., 2019; Tang et al., 2011) report carbon constitutes 11.1%–12.7% of scallop, mussel, oyster, and clam shells' dry weight (a C_f_ value of 0.111–0.127). For our calculations we used a slightly wider range (10%–15% or a C_f_ value of 0.1–0.15) which has previously been reported for green‐lipped mussel shells (Stenton‐Dozey & Broekhuizen, 2019).

In order to determine annual change in shell weight (W), it was necessary to first establish shell growth rates for green‐lipped mussels. McLeod et al. (2012) showed average adult green‐lipped mussel shell length increased 0.04 mm per day on the seafloor of the Hauraki Gulf, which we extrapolated to 14.6 mm per year (a growth rate similar to reports from unpublished theses, ranging from 11.7 to 15.3 mm per year; Van Kampen, 2017; Wilcox, 2017). We used linear regression to create a model that predicts adult green‐lipped mussel shell weight from a given shell length (Figure S1 in the supplement), and, assuming an adult growth rate of 14.6 mm per year, we estimated an average annual increase in shell weight (g) given known shell lengths. We multiplied this value by 15 to estimate the weight increase of our hypothetical mussel clump (m^2^) on an annual basis.

Note that Equation (2) does not quantify total carbon stored in mussel shells (only carbon captured as a result of adult growth), as we are concerned with carbon effects post‐restoration. The majority of growth (and therefore carbon capture in shells) occurs in the first few years of development and decreases significantly with mussel size (Hickman, 1979), and is therefore not accounted for here. We note here that dissolution processes related to shell material are not presented in these calculations, and resulting implications for the budget are further considered in the discussion.

### 2.3 Ecosystem metabolism

Mussel bed ecosystem metabolism was determined through flux measurements capturing the net effects of respiration (CO_2_ effluxes from both bivalves and the benthic communities they support) and local biodeposit remineralization versus carbon fixation (CO_2_ captured by photosynthetic macroalgae, microphytobenthos, and biofilms on shell surfaces). Less time consuming and costly than measuring carbon fluxes directly, oxygen demand was converted to a carbon mineralization or uptake rate (e.g., Clavier, 1994; Oviatt et al., 1986) using respiratory and photosynthetic quotients, respectively.

In February of 2017 and March–April of 2019, in‐situ benthic flux chambers (0.25 × 0.25 m; volume = 41 L) were placed over mussel clumps and control sediments devoid of mussels (~5 m away) at a total of nine different restoration sites in Mahurangi Harbour and Kawau Bay (Table S1) to determine net oxygen fluxes. This was done both under dark and light conditions achieved by covering and uncovering chambers with black polyethylene (methods adapted from Lohrer et al., 2004). Briefly, water samples were taken at the beginning and end of the incubation period (~4 h) and analyzed for O_2_ concentration using a quadrupole membrane inlet mass spectrometer (with Pfeiffer Vacuum Prisma Plus QMG220 M1 QMS, Bay Instruments); these oxygen measurements were checked for congruence with values obtained from miniDOT optical oxygen loggers (Precision Measurement Engineering) placed within the chamber space. With known incubation times for each chamber, fluxes were calculated as the difference between initial and final concentrations and corrected for volume and surface area of the chamber to obtain a flux in μmol m^−2^ h^−1^ (e.g., O'Meara et al., 2020). Oxygen flux data were combined from two published studies (total chambers inside beds = 49, total chambers outside beds = 39; Hillman et al., 2021; Sea et al., 2021) and converted to a carbon mineralization rate using respiratory quotients of 1 and 1.2 under dark conditions, and photosynthetic quotients of 1 and 1.2 under light conditions (deemed acceptable for photic, oligotrophic marine sediments; Clavier, 1994, Carlsson et al., 2010; Attard et al., 2020). Finally we assumed a 12:12 h light cycle and multiplied carbon fluxes from light and dark chambers accordingly to obtain the net carbon ecosystem metabolism over a diel cycle (μmol m^−2^ day^−1^), achieved by subtracting carbon uptake during daylight hours from carbon effluxes during darkness. This net value was converted to grams of carbon per year to achieve units comparable to the rest of the carbon budget.

### 2.4 Sediment carbon storage and consumption

The amount of carbon stored in mussel bed sediments is dependent on local sedimentation rates, biodeposition rates (which also vary with the quality and quantity of organic material filtered and eventually digested/egested), and the rate of carbon degradation at various sediment depths. Each of these ideas is explored below.

#### 2.4.1 Carbon from biodeposition

Using a laboratory experiment, we determined the amount of carbon available to surficial sediments resulting from mussel biodeposition. Our experiment used adult green‐lipped mussels from five restoration sites collected in winter 2017 (Table S1). Mussels were allowed to acclimatize to laboratory conditions for 3 days in 10 L aerated tanks (seawater flow‐through system, filtered on 200 μm mesh; average water temperature 17°C; 10:14 h light cycle). On the day of the experiment, sets of three mussels each were placed in a smaller tank (3 L) and allowed a 1‐hour filtration period in seawater obtained from one of two Mahurangi water sources with contrasting suspended sediment concentrations (either “high” or “low”; 66.67 ± 1.5 or 13.83 ± 1.9 FNU, respectively), ensuring that trials encompassed a range of natural suspended sediment concentrations typically experienced within the Mahurangi Harbour and Kawau Bay. Biodeposits were collected at the end of the filtration period, dried at 60°C for 48 h and weighed. Biodeposition values obtained from mussels filtering each water source (high: *n* = 15; low: *n* = 15) were multiplied by 5 (to upscale to our 15 mussel scenario), and then converted to a biodeposition rate in g m^−2^ year^−1^. Finally, this value was multiplied by the average carbon fraction (6.4%) of mussel biodeposits (Filgueira et al., 2019; Giles & Pilditch, 2004, 2006) to obtain an estimated carbon production rate in g C m^−2^ year^−1^.

#### 2.4.2 Biodeposit degradation rates

Only a portion of carbon in biodeposits is subducted into marine sediments. Others have shown that mussel biodeposits rapidly degrade, and a portion of resultant organic carbon is remineralized (Carlsson et al., 2010; Giles & Pilditch, 2006). Note that the biodeposits of green‐lipped mussels have been shown to exhibit a half‐life of 4.3 days under similar temperature conditions (Giles & Pilditch, 2006), but we do not directly calculate remineralization rates here, as increases in sediment oxygen demand (and subsequent CO_2_ effluxes) resulting from the degradation of biodeposits are included in net fluxes derived from chamber experiments outlined in Section 2.3 above.

#### 2.4.3 Carbon consumption in mussel bed sediments

Finally, not all carbon drawn down to the seafloor is sequestered, as a portion of this carbon fuels microbial and macrofaunal activity in local sediments; resultant bioturbation can further influence sediment organic matter degradation rates and carbon remineralization efficiency (Arndt et al., 2013; Burdige, 2007). We trialed the application of a rapid organic matter assay (ROMA; methods described in O'Meara et al., 2018) to compare carbon consumption rates at different sediment depths. Briefly, two assay plates (with a series of machined wells corresponding to sediment depths of 1, 3, 7, 10, and 15 cm) were filled with a carbon/agar solution, allowed to solidify, and carefully inserted into mussel bed sediments using SCUBA. Plates were left out for a total of 2 weeks at five restoration sites (Table S1) and carbon degradation at various depths determined by calculating the net difference in agar volume over the experiment duration. These methods were repeated in soft‐sediment control locations ~5 m away from restoration sites. ROMA plates were not recovered from the Ngaio Bay control, leaving a total of four control sites used for comparison purposes. Carbon degradation rates generally decrease with depth (O'Meara et al., 2018), and our goal was to determine how relative rates vary with increasing depth across sites and treatments (mussel beds vs. control sediments), and to establish if a “sequestration depth” (consumption rate <1 g C m^−2^ day^−1^) was apparent under the given experimental conditions.

#### 2.4.4 Visualizing carbon storage at various depths

To visualize long‐term changes in carbon storage as a result of mussel restoration, triplicate sediment cores (5 cm diameter, 30 cm length) were taken inside and outside (>5 m away) of four restored beds in late January and early February of 2021 (Table S1). Samples were split into depth segments which concentrated sampling efforts on the uppermost sediment layers (every centimeter for the first 5 cm, then aliquots taken from 5–10 and 10–20 cm) because restored beds had only been in place for a few years. Samples were dried at 60°C, ground with mortar and pestle, and stored in a desiccator prior to analysis. Percent total carbon content of dried sediment samples was determined using a CHSN elemental analyzer (Vario EL Cube, Elementar, Langenselbold, Germany), with results converted to g carbon using known sample weights and later scaled to 1 m^2^. Two‐way ANOVA was used to determine if total carbon content varied by restoration site and status (inside bed or outside bed). Values were log‐transformed to meet assumptions of normality and homogeneity of variance. We adjusted *p*‐values using Holm's Sequential Bonferroni Procedure to control the familywise error rate for multiple hypothesis tests. Significance levels were set to *α* < .05, and standard errors (SEs) were used to assess the precision of mean values. All analyses were conducted using the R statistical package (version 4.1.0).

## 3 RESULTS

### 3.1 Shell formation and dissolution

Our regression model accurately predicted adult green‐lipped mussel shell weight from shell length (*R* ^2^ = 0.782, *p* < .0001; Figure S1), and, assuming a growth rate of 14.6 mm per year, we estimated the weight of an adult mussel shell increases by 11 to 12 g annually. Using our 15 mussel m^2^ model system, this becomes an additional 165–180 grams per year (term W in Equation 2). Given the accepted carbon fraction range for green‐lipped mussels (C_f_ = 0.1–0.15), we estimated carbon storage between 16.5 and 27.0 g C m^−2^ year^−1^. This degree of calcium carbonate precipitation results in CO_2_ emissions totaling ~9.9 to 16.2 g C m^−2^ year^−1^, or total net storage of −10.8 to −6.6 g C m^−2^ year^−1^ as a combined result of shell formation and dissolution processes (Figure 2a).

### 3.2 Ecosystem metabolism

**FIGURE 3:**
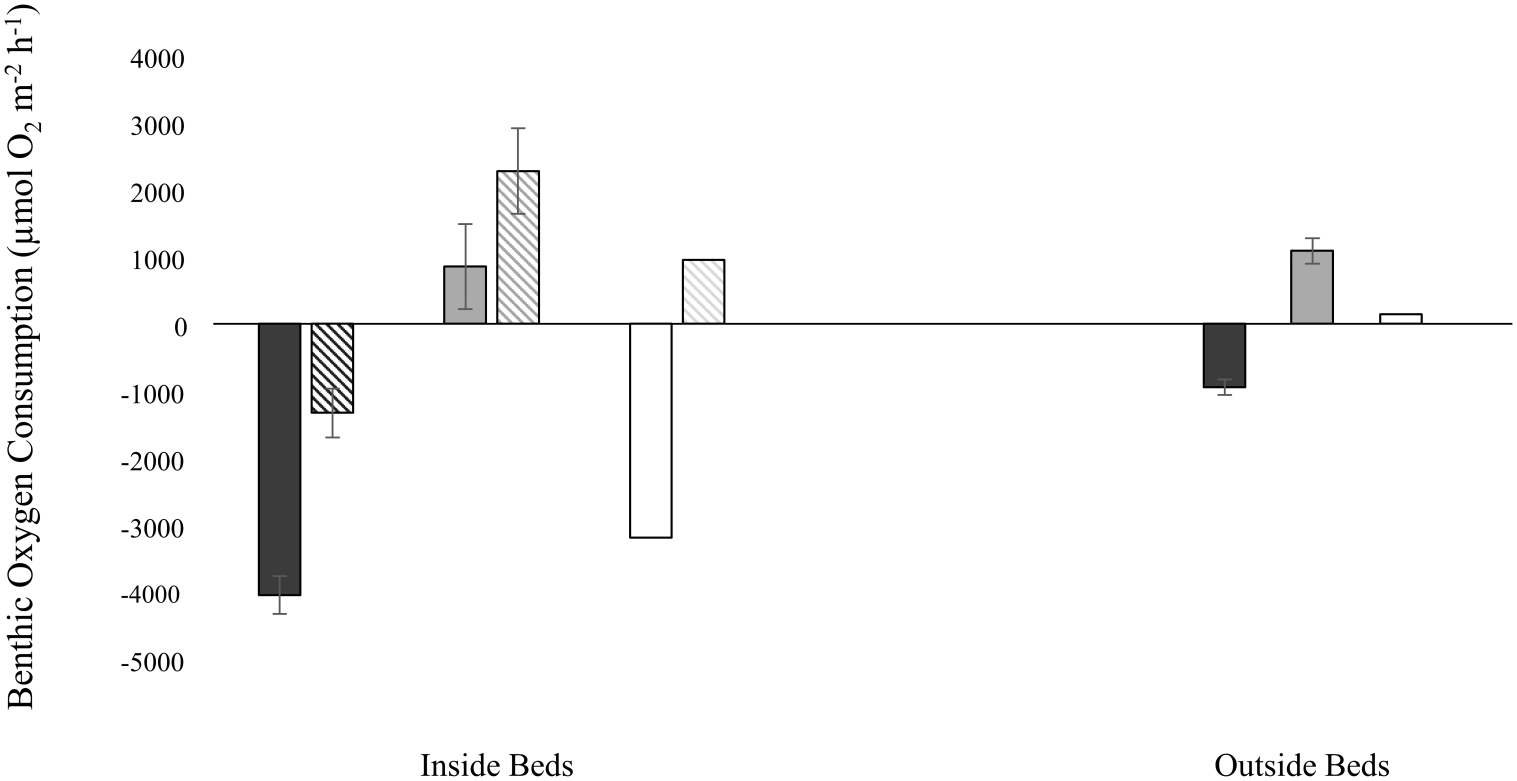
Results of benthic chamber experiments in nine restored mussel beds, comparing average benthic oxygen consumption inside and outside (>5 m away) of mussel beds, under light and dark conditions (grey and black bars, respectively). For visual reference, white bars show the calculated net oxygen flux (difference between light and dark chambers). Diagonal bars inside beds represent sediment oxygen demand after removing the direct effect of mussel respiration on measured O_2_ fluxes from Hillman et al. (2021) (oxygen demand of 15 mussels determined using regression equation in Figure S3).

We recorded net oxygen consumption in all benthic chambers under dark conditions within mussel beds, with an average uptake rate of −4040.4 ± 284.0 (SE) μmol O_2_ m^−2^ h^−1^ (Figure 3). Even with large heterotrophic bivalves in the chamber space, an average net oxygen efflux was observed under light conditions (857.0 ± 636.8 μmol O_2_ m^−2^ h^−1^), although these chambers exhibited roughly twice as much variation as their dark counterparts. Using the accepted range of respiratory and photosynthetic quotients resulted in a carbon mineralization rate within mussel beds of 4040.4 ± 284.0 to 4848.4 ± 340.8 μmol CO_2_ m^−2^ h^−1^ in the dark, and carbon uptake between −714.2 ± 530.6 and −857.0 ± 636.8 μmol CO_2_ m^−2^ h^−1^ in the light. Over a diel cycle this becomes a net carbon efflux of 0.459–0.595 g C m^−2^ day^−1^, or an annual efflux of 167.6–217.2 g C m^−2^ year^−1^ (Figure 2b).

In contrast, dark control sediments outside mussel beds had an average oxygen consumption rate roughly four times less than that of dark sediments within mussel beds (−940.6 ± 115.7 μmol O_2_ m^−2^ h^−1^). Average net oxygen production was again observed in light chambers outside mussel beds (1087.0 ± 191.8 μmol O_2_ m^−2^ h^−1^), a rate comparable to (but slightly greater than) those reported within beds (Figure 3). These oxygen consumption and production rates in control sediments result in carbon mineralization and fixation rates of 940.6 ± 115.7 to 1128.7 ± 138.9 μmol CO_2_ m^−2^ h^−1^ and ‐905.9 ± 159.9 to −1087.0 ± 191.8 μmol CO_2_ m^−2^ h^−1^, respectively. Corresponding net diel ecosystem metabolism in control sediments overlaps with zero (from carbon uptake of −0.021 g C m^2^ d^−1^ to a carbon efflux of 0.032 g C m^2^ d^−1^), equating to an annual carbon emission/uptake range of −7.7 to +11.7 g C m^−2^ year^−1^ in sediments devoid of mussels.

### 3.3 Sediment carbon storage and consumption

Nearly twice as many biodeposits were produced at higher concentrations of suspended solids (two‐way ANOVA; *F*_1,24_ = 27.8; *p* < .0001; Figure S2). We averaged the biodeposit production rates across all five sites for each water source (high vs. low suspended solid concentration) to estimate that each clump of three mussels produces between 0.0110 ± 0.0007 and 0.0202 ± 0.0017 g biodeposits h^−1^. Extrapolated to the idealized mussel clump scale on an annual basis, biodeposition rates were estimated between 483.0 and 883.9 g biodeposits m^−2^ year^−1^. Utilizing the average carbon content of mussel biodeposits (6.41%) results in additional carbon draw‐down (−) due to benthic–pelagic coupling of 31.0 to 56.7 g C m^−2^ year^−1^ to the seafloor (Figure 2c).

**FIGURE 4:**
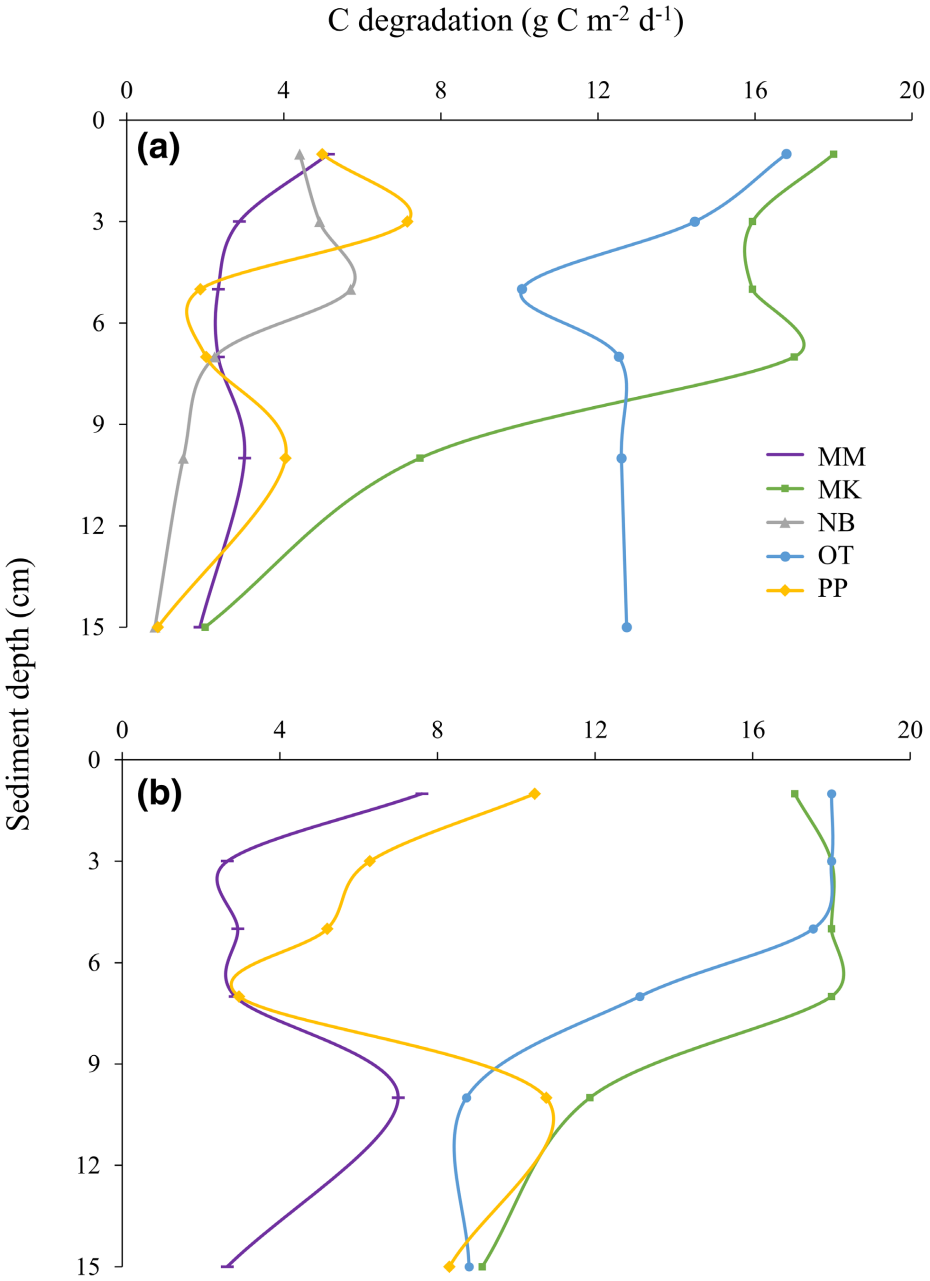
Calculated carbon degradation rates at various sediment depths using rapid organic matter assay (ROMA) plates in (a). subtidal restored mussel beds, and (b). control sediments devoid of mussels (~5 m away from restoration sites). Site labels: MM, Mahu Mid; MK, Motoketekete; NB, Ngaio Bay; OT, Otarawao Bay; PP, Pukapuka.

Carbon degradation rates calculated from rapid organic matter assays decreased with depth at all five restoration sites, but both the rate and magnitude of change in degradation appeared to vary by site (Figure 4). Mussel beds within Mahurangi Harbour (MM, NB, PP) had surface degradation rates approximately three times lower than beds within (or near the entrance of) Kawau Bay. At control sites, greater carbon degradation rates were similarly observed in sandier Kawau Bay sediments, although notable increases in calculated degradation rates were observed in muddier, Mahurangi Harbour control sediments at 10 cm depth. With the exception of site OT, all mussel beds converged on carbon degradation rates between ~1 and 3 g C m^−2^ day^−1^ at the deepest sediment depth tested (15 cm). At this same depth, carbon degradation rates were roughly two‐ to threefold higher in control sediments. Comparing each mussel bed to its control sediments revealed a general pattern of increased degradation activity in soft‐sediment controls, a pattern which continued down to depths of 15 cm at all but one site (OT; Figure 4).

**FIGURE 5:**
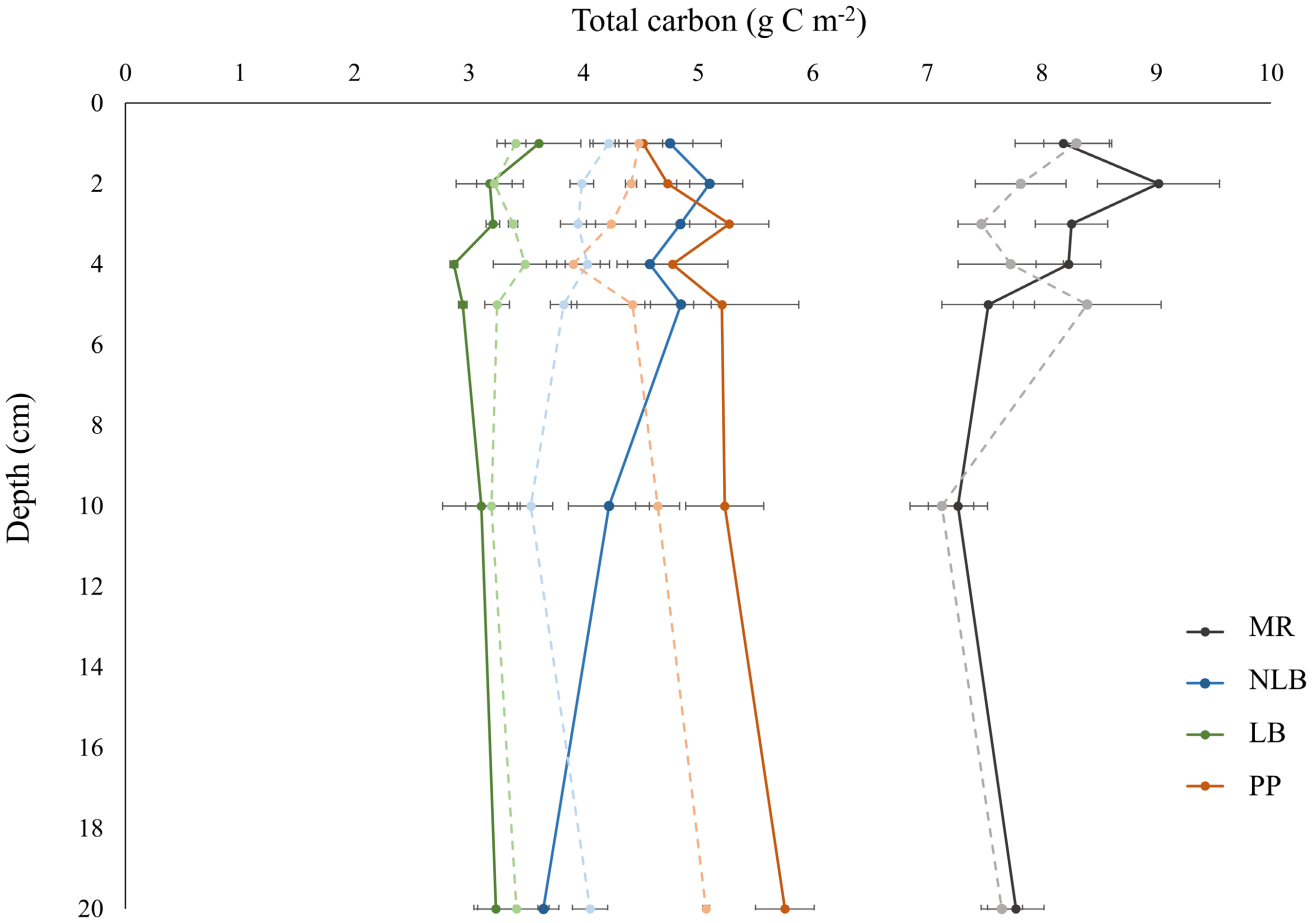
Total carbon from sediment cores, separated by depth at mussel restoration sites (solid lines) and nearby sediments (>5 m away) devoid of mussels (dashed lines). Site labels: MR, Motuora; NLB, New Lagoon Bay; LB, Lagoon Bay; PP, Pukapuka.

While carbon degradation rates were typically highest at the sandier, Kawau Bay sites, total carbon storage determined from sediment cores was also noticeably greater (roughly two times that of muddier beds; Figure 5). Increased total carbon content was also observed in sediment cores taken outside the mussel bed at Motuora, and significant site differences in carbon content were detected at all sediment depths tested (Table S2). Mussel beds did not have a significant effect on the amount of carbon recorded in surficial sediments (0–1 cm; two‐way ANOVA; *F*_1,20_ = 1.8; *p* = .785), but appeared to make notable contributions just below the sediment surface (depths of 1–2 and 2–3 cm; two‐way ANOVA; *F*_1,19_ = 7.0; *p* = .093 and two‐way ANOVA; *F*_1,19_ = 9.6; *p* = .041, respectively). With the exception of Lagoon Bay, total carbon measured in mussel beds increased for the first few centimeters of sediment, and then gradually decreased with increasing sediment depth, while carbon content at all non‐restored sites initially decreased relative to surface sediments at these depths (Figure 5).

Figure 2 summarizes the overall budget estimated for restored mussel bed sites. A direct summation of biogenic calcification, benthic ecosystem metabolism, and biodeposition processes suggests a moderate carbon efflux (+100.1 to 179.6 g C m^−2^ year^−1^) occurs within restored mussel beds, with bed sediments exhibiting lower carbon degradation rates and higher carbon content than controls. Control sites are considered relatively neutral in terms of their carbon contributions (benthic oxygen consumption resulting in −7.7 to +11.7 g C m^−2^ year^−1^), with sediments generally characterized by higher carbon degradation rates and lower carbon content than restored beds.

## 4 DISCUSSION

Here we combined published literature with experiments to illustrate that shallow, coastal environments dominated by dense bivalve beds are significant zones of carbon transformation contributing to ecosystem processes and community metabolism. Notable differences in carbon cycling occur at restoration sites compared to controls, with mussel beds characterized by greater carbon mineralization rates but generally higher carbon content and lower degradation rates in underlying sediments. We provide a carbon budget for New Zealand's shellfish restoration efforts and report a range of potential carbon effluxes, importantly demonstrating that carbon‐related services/outcomes are affected by the highly heterogeneous nature of coastal ecosystems and selected restoration locations. Similar to other budgets, our work necessarily simplifies complexities inherent in dynamic, coastal systems, but our first‐order approximation represents a current best‐estimate given limited data availability and considers a number of ecological nuances often overlooked in comparable budgets of terrestrial forests (e.g., Aalde et al., 2006). Our reported carbon effluxes fit well within the accepted range of fluxes published in other studies balancing biogenic calcification and respiration against carbon sequestration in other mussel species (from a net sink of 7.6 g C m^−2^ year^−1^ to a net source of 1656 g C m^−2^ year^−1^; Hily et al., 2013; Mistri & Munari, 2013; Munari et al., 2013; Filgueira et al., 2019) and utilizes a holistic approach to create ecologically meaningful conclusions regarding the role of bivalves in carbon cycling.

### 4.1 Shell formation/dissolution

Biogenic calcification provides a modest form of carbon storage in restored mussel bed systems (Figure 2a). However, our estimates are highly conservative owing to the wide range of possible C_f_ values for green‐lipped mussels; the carbon fraction of mussel shell's dry weight is reported to be above 12% for other mussel species (van der Schatte Olivier et al., 2020), and refining this value for green‐lipped mussels would better reflect (and likely increase) the carbon sink capacity of restored beds. In addition our methods do not consider carbon stored in mussels before utilization in restoration projects; the majority of growth occurs in the first 2 years of life, with average juvenile growth rates three times higher than what we report for adult mussels in this study (Hickman, 1979). If restored beds begin to self‐recruit in the future, we could credit additional carbon storage occurring over the first few years of rapid development, potentially doubling our estimates of carbon in shell material. In the current absence of recruitment, however, restoration projects solely utilizing juvenile mussels (< 30 mm shell length) have been trialed with limited success as a likely consequence of local predation pressures (Alder et al., 2021). Restoration in New Zealand will likely move forward using adult mussels, and until recruitment occurs there is little additional capacity for carbon storage through the process of shell formation.

It is also important to note that carbon is only temporarily stored in a living mussel's shell; the fate of this carbon depends on local environmental conditions (hydrodynamic forces, sediment conditions, local sedimentation rates, etc.), the combination of which ultimately determines if shell is buried and stored deep within the sediment before it is allowed to dissolve (e.g., DeAlteris, 1988; Fodrie et al., 2017). Green‐lipped mussel shells are thin and brittle, and previous studies (even preventing physical abrasion and mechanical breakdown of shell) within the Hauraki Gulf report dissolution rates of 5%–8% of mussel shell weight within 300–500 days (Smith, 1992). Furthermore, calcium carbonate actively breaks down in the uppermost layers of bioturbated sediments (Waldbusser et al., 2011), and, as pore water acidity rapidly increases within the first few centimeters of sediment in the Mahurangi Harbour, it is unlikely significant amounts of carbon captured in green‐lipped mussel shells are stored below surficial sediment depths at present (and the modest flux presented in part A of the budget could be closer to a net neutral contribution if shells grow and dissolve at a similar rate). We note, however, that the bioengineering capabilities of mussels might eventually allow beds to alter sediment conditions over longer time frames, encouraging the storage of shell material if sediments become well‐oxygenated and exhibit higher porosity in the future. In light of current sediment conditions, those interested in tracing CO_2_ emissions resulting from biogenic calcification might also note that if shell material is allowed to dissolve in seawater, ensuing alkalinity changes to the carbonate system will actually result in equivalent atmospheric CO_2_ concentrations prior to shell formation. To simplify these dynamics we have chosen to examine carbon storage on a similar timescale used in terrestrial forest budgets and report a downwards flux (−) of carbon as a result of biogenic calcification, but acknowledge that the long‐term, inorganic carbon storage capacity of these systems is subject to a multitude of processes (local aerobic respiration, feedbacks with calcifiers, freshwater discharge, and anthropogenic CO_2_ emissions) that ultimately influence the stability of carbonates in estuarine environments (Abril et al., 2003; Feely et al., 2002; Miller et al., 2009; Salisbury et al., 2008). We currently suggest that restoration practitioners interested in improving the carbon burial potential of restoration projects utilize calcium carbonate producing organisms with thicker shells less prone to dissolution (ideally with high C_f_ values), and consider restoration sites exhibiting less acidic sediment conditions (e.g., lower concentrations of organic material and well oxygenated, high porosity sediments). Initial assessments which verify the compatibility of environmental conditions with pre‐determined restoration objectives will prove to be invaluable and will inform both the location and species most suitable for achieving desired ecosystem service outcomes.

Additionally, greenhouse gas projections indicate that the role of bivalve restoration projects in coastal carbon cycling will likely change under future climate change scenarios. Further oceanic uptake of anthropogenic carbon will alter seawater carbonate chemistry and reduce the ability of mussels to calcify, while changes in sea surface temperature will impact physiological processes outlined in our budget (e.g., Parker et al., 2013; Resgalla Jr et al., 2007; Talmage & Gobler, 2010). Together these combined effects will influence biogeochemical cycles, ecosystem functioning, and the magnitude of services provided by bivalve‐dominated systems. Restoration practitioners must be proactive in selecting species and sites which maximize the probability of ecosystem service delivery under future climate conditions.

### 4.2 Ecosystem metabolism

Results of net ecosystem metabolism experiments in mussel beds mirror those of others (Attard et al., 2020; Chauvaud et al., 2003; Lejart et al., 2012; Munari et al., 2013) who report bivalve respiration rates exceed rates of primary production and/or storage in shell material and consistently result in net carbon effluxes from the sediment (Figures 2 and 3). In contrast to blue carbon habitats which support numerous faunal species and still contribute to net carbon draw‐down, it seems unlikely that the additional contribution of large, heterotrophic bivalves at our restoration sites (also known to support abundant benthic communities which consequently respire further CO_2_), could be offset by local autotrophic communities (oxygen‐producing biofilms, macroalgae, microphytobenthos; Heisterkamp et al., 2013, Rodil et al., 2019). Despite their heterotrophic status, bivalve beds—by enhancing suitable substrate and nutrient availability—are increasingly acknowledged for their ability to support notable communities of carbon‐fixing primary producers (Attard et al., 2019; Norling & Kautsky, 2007; Volaric et al., 2018) and can achieve gross primary production rates comparable to nearby vegetated coastal habitats (Attard et al., 2019; Attard et al., 2020). The additional role of bivalves in lowering turbidity through sediment stabilization and filtration processes has also been shown to promote primary productivity (Newell et al., 2005; Newell & Koch, 2004). Previous studies at green‐lipped mussel restoration sites similarly indicate local enhancement of gross primary production through increased abundance of primary producing communities relative to nearby control sediments (Sea et al., 2022a; Sea et al., 2022b). Physical and biological alterations in the restoration environment are favorable to carbon‐fixing autotrophs and can therefore result in additional sources of autochthonous, labile carbon significant to the overall influence of mussel restoration in mediating biogeochemical cycles.

It is noteworthy that other studies (Attard et al., 2020; Rodil et al., 2019) attribute as much as half of reported carbon turnover in mussel beds to microbial species supported by beds. As some carbon accounting studies related to shellfish aquaculture call for separate carbon budgets regarding shell formation and tissue growth (a by‐product vs. the portion valued for human consumption, e.g., Filgueira et al., 2015), this motivates theoretical questions regarding the appropriateness of dissociating the respiratory needs of heterotrophic bivalves from their effects on overall ecosystem metabolism (as our current report largely showcases an efflux inevitable of any primary consumer used in restoration efforts). Here we estimate the average oxygen demand of an adult, green‐lipped mussel to be 94.8 μmol O_2_ h^−1^ (Figure S3). Mussel respiration therefore accounts for a significant portion (an additional 1422 μmol O_2_ h^−1^) of the calculated carbon mineralization rate in benthic chambers under dark conditions; after removing the direct effect of mussel respiration on measured fluxes, base‐line oxygen demand is comparable to nearby bare sediments (Figure 3). As we determined the oxygen requirements of 15 mussels m^−2^ to be substantial, it is notable that net fluxes observed under light conditions were similar inside and outside of beds (Figure 3). Achieving a similar net sediment oxygen demand under light conditions is indicative of enhanced primary productivity within mussel beds.

### 4.3 Sediment carbon storage/consumption

To the best of our knowledge this work curates all previous attempts to document carbon‐related changes in the sediment environment as a result of green‐lipped mussel introductions, but we recognize that ideas related to sediment carbon storage and consumption (part C in Figure 2) are less well defined/constrained than other processes outlined in this text, attributable to the timescale of our studies and the complex, heterogeneous nature of coastal soft sediments. We utilized data from mussel beds under varying hydrodynamic and edaphic conditions to help scale our findings while encompassing natural variability, and can conclude at present that carbon storage is not comparable to typical blue carbon habitats (e.g., Duarte et al., 2005). Research efforts which further resolve related spatial and temporal heterogeneity in the sediment environment will make notable contributions in refining the carbon budget reported here.

Adult green‐lipped mussels are known to alter feeding behavior (filtration and rejection rates) in response to seston quality and quantity, although these rates generally increase at higher concentrations of organic matter in the water column (Hatton et al., 2005). By sourcing water of contrasting quality this additional source of variation was incorporated into our study design, and resulting biodeposition rates are in general agreement with other mussel studies (Attard et al., 2020; Hatton et al., 2005; Hawkins et al., 1999). While pairing increased filtration rates with increased rejection rates of lower‐quality particles proves to be energy efficient for mussels experiencing higher turbidity systems (Bayne et al., 1993; Hatton et al., 2005), it should be noted that, as restored green‐lipped mussel beds continue to filter out large quantities of suspended material from the Hauraki Gulf's water column, the carbon content of biodeposits will likely increase over time (as the proportion of filtered seston including carbon‐fixing phytoplankton increases). However, it is also possible that the ability of mussels to alter feeding behavior may partially negate carbon‐related benefits to the sediment environment as local sedimentation rates may decrease if filtration rates decrease.

Perhaps a subject which warrants further exploration is the quality and fate of locally produced biodeposits. Note that hydrodynamic forces and mussel spatial arrangement vary with restoration location and can result in the removal/transport of available, carbon‐rich biodeposits from local restoration sites (Sea et al., 2022a; Sea et al., 2022b). A current lack of particle tracer studies also precludes the determination of how biodeposits interact with local sediments, as well as what portion of produced biodeposits (estimated at −56.7 to −31.0 g C m^−2^ year^−1^) are subducted to a depth at which available carbon can be considered sequestered. Additionally, studies analyzing biodeposit degradation from other mussel species suggest that, while the labile fraction of deposits is rapidly remineralized, a larger fraction of refractory carbon (as much as 80% of total POC in biodeposits) remains over longer timescales (days to weeks) and is available for potential storage in sediments (Carlsson et al., 2010). Further temporal resolution of biodeposit‐associated carbon degradation and subduction in marine sediments is necessary and will have significant capacity to alter conclusions regarding the ability of restoration projects to sequester carbon.

Rapid organic matter assay trials suggest that the rate of organic carbon degradation varies with location, sediment depth, and restoration status. We note here that others have importantly shown biotic factors (specifically macrofaunal and microbial activity not linked to scale of individual ROMA plates in this study) are the most important drivers of observed carbon degradation rates in coastal marine sediments (O'Meara et al., 2018), and that significant carbon enrichment and alterations to macrofaunal biomass and community composition can occur at the bivalve‐patch scale (Norkko et al., 2001). Global studies illustrate that the average maximum depth at which surface sediments are turned over through bioturbation activities is roughly 5–10 cm (Boudreau, 1998; Teal et al., 2008), and we generally observed a trend in decreased carbon degradation activity below these depths. Although the observed variability in ROMA plate trials precludes our ability to confidently determine a “carbon sequestration depth” here, it appears such a value would likely be realized at shallower sediment depths (perhaps near 15 cm) within restored mussel beds than nearby bare sediments (Figure 4). The bioturbating activities of infauna are typically considered a source of additional oxygen to deeper sediment layers, which generally increases organic matter mineralization in marine sediments (Burdige, 2007). This, in addition to the direct consumption of organic material by infauna, can lead to an overall decrease in sediment organic matter preservation. However, some infaunal species have been shown to play a significant role in the draw‐down of refractory carbon at depth (Levin et al., 1997). We show elsewhere that infaunal community composition changes with mussel restoration site (Sea et al., 2022a; Sea et al., 2022b), and, coupled with the results of carbon cores, suggest that benthic infauna play a more complex role in the preservation of organic matter. Current estimates of carbon degradation and rates of burial within marine sediments vary significantly with space and time (Arndt et al., 2013 and references within), warranting more extensive analysis to resolve the heterogeneity observed within mussel beds. Determining both the decay rates of sediment carbon pools and the depth at which organic carbon is considered “sequestered” are identified as major uncertainties in current carbon accounting efforts (Macreadie et al., 2017) which must be resolved to accurately inform future coastal carbon budgets.

Results from carbon cores (Figure 5) show no significant difference in total carbon content at the approaching “sequestration depth” of 15 cm in mussel bed sediments (established from Figure 4); however, we estimate that sediments collected below this depth were at least 30 years old at the time of study (local sedimentation rate of ~4 mm year^−1^; Oldman et al., 2009) and were therefore incapable of being affected by recent restoration efforts. It is reasonable that differences in carbon content were only detected at depths in which restoration was able to influence the sediment environment (around 1 to 3 cm in depth, or sediments estimated to be from the past 2–7 years at current sedimentation rates). While it is possible that large, bioturbating macrofauna introduce additional non‐refractile carbon to sediment depths greater than what would be predicted through passive sedimentation alone (e.g., Levin et al., 1997; Schenone et al., 2019), such organisms were not observed in partitioned sediment samples. As we measured total carbon content here, forthcoming analyses separating the proportion of inorganic carbon (a fraction which can be differentiated into geogenic and biogenic sources) from organic carbon (which can be further differentiated into autochthonous and allochthonous sources of significance to New Zealand estuaries; Bulmer et al., 2020) will make worthwhile contributions to our understanding of sediment dynamics in restored mussel bed systems.

## 5 CONCLUSION

Our results document how green‐lipped mussel restoration projects affect carbon cycling but also importantly infer that the loss of extensive regional beds (~1500 km^2^) has resulted in major changes in carbon transport and transformation in the Hauraki Gulf. Disturbance of carbon stocks (dredging, deforestation, etc.) can transform systems of net carbon storage into net sources of atmospheric carbon (Donato et al., 2011; Luisetti et al., 2019; Mcleod et al., 2011). And while not directly examined here, mussel beds are known to significantly increase sediment stabilization and cohesion (Widdows & Brinsley, 2002), important in the modern ocean where anthropogenic disturbance of sediments and resulting resuspension reduces organic carbon content by as much as 50% (Pusceddu et al., 2014) and decreases overall carbon turnover and preservation in marine sediments (Keil, 2017). Therefore, conservation and restoration solutions which avoid further degradation, protect natural carbon sinks, and actively create further potential for storage should be given high management priority. Quantification of organic carbon stocks will likely provide justification for the expansion of regional mussel restoration projects and make a case for their protection, as global reef dredging was estimated to have reintroduced over 400,000,000 Mg of carbon into coastal waters (Fodrie et al., 2017) and New Zealand's former shellfisheries (e.g., Paul, 2012) partially responsible for emissions.

## AUTHOR CONTRIBUTIONS

MAS, JRH, and SFT conceptualized the study. MAS and JRH contributed to data collection and analyses. All authors contributed to the interpretation and writing of the manuscript.

## CONFLICT OF INTEREST

The authors declare no competing interests.

## Supporting information


**Appendix S1** Supporting information

## Data Availability

The data that support the findings of this study are openly available in Pangaea at https://doi.org/10.1594/PANGAEA.943087.
