# Supplementary material for: The influence of mussel restoration on coastal carbon cycling
Source: Glob Chang Biol. 2022 Jun 20;28(17):5269–82. doi: 10.1111/gcb.16287 (PMC9544040; doi:10.1111/gcb.16287)

*The following supplement accompanies the article*

**The Influence of Mussel Restoration on Coastal Carbon Cycling**

**Mallory A. Sea*, Jenny R. Hillman, and Simon F. Thrush**

*Corresponding author: msea579@aucklanduni.ac.nz

**Table S1.** List of mussel restoration sites and local sediment characteristics (mean and range) as determined by Sea et al. (2021), Hillman et al. (2021), and Sea et al. (2022). Black squares indicate where data from a particular mussel bed were included in the carbon budget. Mud is comprised of silt + clay (< 63 μm). SOM: sediment organic material.

| Site | Date established | Deployment (tonnes) | Mud content (%) | SOM (%) | Chl a content (µg g-1) | Shell weight vs. length regression | Benthic chambers | Biodeposit experiment | ROMA plates | Carbon cores |
| --- | --- | --- | --- | --- | --- | --- | --- | --- | --- | --- |
| Mahurangi Mid | October 2016 | 1 | 26.1  (23.3-30.3) | 3.1  (2.7-3.5) | 21.1  (20.4-21.8) | ■ | ■ | ■ | ■ |  |
| Martins Bay N | October 2016 | 1 | 14.2  (10.6-19.5) | 2.8  (2.3-3.4) | 6.7  (6.3-7.2) |  | ■ |  |  |  |
| Martins Bay S | October 2016 | 1 | 6.7  (5.3-9.7) | 2.0  (1.7-2.5) | 5.4  (4.4-6.4) |  | ■ |  |  |  |
| Motuora | November 2017 | 10 | 7.0  (4.2-10.4) | 3.4  (2.6-4.8) | 4.9  (2.9-6.2) | ■ | ■ | ■ |  | ■ |
| Motoketekete | October 2016 | 1 | 7.5  (6.4-8.7) | 2.0  (1.8-2.1) | 8.6  (8.2-8.9) |  | ■ |  | ■ |  |
| Ngaio Bay | October 2016 | 1 | 50.2  (39.9-59.1) | 4.9  (4.6-5.2) | 9.1  (8.8-9.5) | ■ | ■ | ■ | ■ |  |
| New Lagoon Bay | July 2019 | 20 | 24.2  (14.6-44.1) | 5.9  (4.1-8.0) | 10.9  (5.2-14.0) |  |  |  |  | ■ |
| Lagoon Bay | November 2018 | 10 | 22.9  (20.2-25.8) | 2.7  (2.0-3.4) | 5.6  (1.6-7.9) |  | ■ |  |  | ■ |
| Otarawao Bay | October 2016 | 1 | 24.5  (20.2-28.1) | 3.2  (2.6-3.9) | 13.0  (12.7-13.3) | ■ | ■ | ■ | ■ |  |
| Pukapuka | November 2018 | 10 | 32.2  (28.3-35.8) | 3.9  (3.3-4.3) | 9.4  (7.6-11.3) | ■ | ■ | ■ | ■ | ■ |

**Table S2.** Results of 2-way ANOVA showing the effects of Site and Status (mussel bed vs. bare sediment) on total carbon content at different sediment depths. Analyses performed on transformed data to meet normality assumptions. P-values were adjusted using Holm’s sequential Bonferroni procedure. Site labels: Pukapuka = PP, Lagoon Bay = LB, New Lagoon Bay = NLB, and Motuora = MR.

* = p < 0.1, ** = p < 0.5, *** = p <0.01

| **Factor** | df | SS | MS | F value | p-value |
| --- | --- | --- | --- | --- | --- |
| **Depth: 0-1 cm** |  |  |  |  |  |
| Site | 3 | 3.063 | 1.021 | 62.865 | < 0.001^***^ |
| Status | 1 | 0.030 | 0.030 | 1.821 | 0.785 |
| Residuals | 20 | 0.325 | 0.016 |  |  |
|  |  |  |  |  |  |
| Pairwise comparison for site (Tukey) | | Diff in Means | Lower confidence interval | Upper confidence interval | Adjusted p-value |
| NLB-MR |  | -0.668 | -0.866 | -0.469 | < 0.001^***^ |
| LB-MR |  | -0.913 | -1.111 | -0.714 | < 0.001^***^ |
| PP-MR |  | -0.663 | -0.861 | -0.464 | < 0.001^***^ |
| LB-NLB |  | -0.245 | -0.451 | -0.039 | 0.016^**^ |
| PP-NLB |  | 0.005 | -0.201 | 0.211 | 0.999 |
| PP-LB |  | 0.250 | 0.044 | 0.456 | 0.014^**^ |
|  |  |  |  |  |  |
|  |  |  |  |  |  |
| **Factor** | df | SS | MS | F value | p-value |
| **Depth: 1-2 cm** |  |  |  |  |  |
| Site | 3 | 2.908 | 0.970 | 95.849 | < 0.001^***^ |
| Status | 1 | 0.071 | 0.071 | 7.049 | 0.093^*^ |
| Residuals | 19 | 0.192 | 0.010 |  |  |
|  |  |  |  |  |  |
|  |  |  |  |  |  |
| Pairwise comparison for site (Tukey) | | Diff in Means | Lower confidence interval | Upper confidence interval | Adjusted p-value |
| NLB-MR |  | -0.621 | -0.784 | -0.458 | < 0.001^***^ |
| LB-MR |  | -0.967 | -1.130 | -0.804 | < 0.001^***^ |
| PP-MR |  | -0.606 | -0.769 | -0.443 | < 0.001^***^ |
| LB-NLB |  | -0.346 | -0.509 | -0.183 | < 0.001^***^ |
| PP-NLB |  | 0.015 | -0.148 | 0.178 | 0.993 |
| PP-LB |  | 0.361 | 0.198 | 0.524 | < 0.001^***^ |
|  |  |  |  |  |  |
|  |  |  |  |  |  |
| **Factor** | df | SS | MS | F value | p-value |
| **Depth: 2-3 cm** |  |  |  |  |  |
| Site | 3 | 2.358 | 0.786 | 93.745 | < 0.001^***^ |
| Status | 1 | 0.081 | 0.081 | 9.612 | 0.041^**^ |
| Residuals | 19 | 0.159 | 0.008 |  |  |
|  |  |  |  |  |  |
| Pairwise comparison for site (Tukey) | | Diff in Means | Lower confidence interval | Upper confidence interval | Adjusted p-value |
| NLB-MR |  | -0.587 | -0.736 | -0.438 | < 0.001^***^ |
| LB-MR |  | -0.868 | -1.017 | -0.720 | < 0.001^***^ |
| PP-MR |  | -0.510 | -0.659 | -0.362 | < 0.001^***^ |
| LB-NLB |  | -0.281 | -0.430 | -0.132 | < 0.001^***^ |
| PP-NLB |  | 0.077 | -0.072 | 0.225 | 0.483 |
| PP-LB |  | 0.358 | 0.209 | 0.507 | < 0.001^***^ |
|  |  |  |  |  |  |
|  |  |  |  |  |  |
| **Factor** | df | SS | MS | F value | p-value |
| **Depth: 3-4 cm** |  |  |  |  |  |
| Site | 3 | 2.715 | 0.905 | 57.818 | < 0.001^***^ |
| Status | 1 | 0.014 | 0.015 | 0.926 | 0.999 |
| Residuals | 19 | 0.297 | 0.016 |  |  |
|  |  |  |  |  |  |
| Pairwise comparison for site (Tukey) | | Diff in Means | Lower confidence interval | Upper confidence interval | Adjusted p-value |
| NLB-MR |  | -0.618 | -0.821 | -0.415 | < 0.001^***^ |
| LB-MR |  | -0.926 | -1.129 | -0.723 | < 0.001^***^ |
| PP-MR |  | -0.617 | -0.821 | -0.414 | < 0.001^***^ |
| LB-NLB |  | -0.308 | -0.511 | -0.104 | 0.002^***^ |
| PP-NLB |  | 0.001 | -0.202 | 0.204 | 0.999 |
| PP-LB |  | 0.308 | 0.105 | 0.511 | 0.002^***^ |
|  |  |  |  |  |  |
|  |  |  |  |  |  |
| **Factor** | df | SS | MS | F value | p-value |
| **Depth: 4-5 cm** |  |  |  |  |  |
| Site | 3 | 2.737 | 0.912 | 44.189 | < 0.001^***^ |
| Status | 1 | 0.014 | 0.014 | 0.681 | 0.999 |
| Residuals | 19 | 0.392 | 0.021 |  |  |
|  |  |  |  |  |  |
| Pairwise comparison for site (Tukey) | | Diff in Means | Lower confidence interval | Upper confidence interval | Adjusted p-value |
| NLB-MR |  | -0.611 | -0.844 | -0.377 | < 0.001^***^ |
| LB-MR |  | -0.941 | -1.175 | -0.708 | < 0.001^***^ |
| PP-MR |  | -0.515 | -0.748 | -0.282 | < 0.001^***^ |
| LB-NLB |  | -0.331 | -0.564 | -0.097 | 0.004^***^ |
| PP-NLB |  | 0.096 | -0.138 | 0.329 | 0.663 |
| PP-LB |  | 0.426 | 0.193 | 0.659 | < 0.001^***^ |
|  |  |  |  |  |  |
|  |  |  |  |  |  |
| **Factor** | df | SS | MS | F value | p-value |
| **Depth: 5-10 cm** |  |  |  |  |  |
| Site | 3 | 2.308 | 0.769 | 59.528 | < 0.001^***^ |
| Status | 1 | 0.028 | 0.028 | 2.171 | 0.785 |
| Residuals | 19 | 0.246 | 0.013 |  |  |
|  |  |  |  |  |  |
| Pairwise comparison for site (Tukey) | | Diff in Means | Lower confidence interval | Upper confidence interval | Adjusted p-value |
| NLB-MR |  | -0.625 | -0.810 | -0.441 | < 0.001^***^ |
| LB-MR |  | -0.833 | -1.018 | -0.649 | < 0.001^***^ |
| PP-MR |  | -0.380 | -0.564 | -0.195 | < 0.001^***^ |
| LB-NLB |  | -0.208 | -0.392 | -0.023 | 0.024^**^ |
| PP-NLB |  | 0.245 | 0.061 | 0.430 | 0.007^***^ |
| PP-LB |  | 0.453 | 0.269 | 0.638 | < 0.001^***^ |
|  |  |  |  |  |  |
|  |  |  |  |  |  |
| **Factor** | df | SS | MS | F value | p-value |
| **Depth: 10-20 cm** |  |  |  |  |  |
| Site | 3 | 2.566 | 0.855 | 106.265 | < 0.001^***^ |
| Status | 1 | 0.000 | 0.000 | 0.004 | 0.999 |
| Residuals | 19 | 0.153 | 0.008 |  |  |
|  |  |  |  |  |  |
| Pairwise comparison for site (Tukey) | | Diff in Means | Lower confidence interval | Upper confidence interval | Adjusted p-value |
| NLB-MR |  | -0.696 | -0.841 | -0.550 | < 0.001^***^ |
| LB-MR |  | -0.848 | -0.994 | -0.702 | < 0.001^***^ |
| PP-MR |  | -0.356 | -0.502 | -0.211 | < 0.001^***^ |
| LB-NLB |  | -0.152 | -0.298 | -0.007 | 0.038^**^ |
| PP-NLB |  | 0.339 | 0.193 | 0.485 | < 0.001^***^ |
| PP-LB |  | 0.492 | 0.346 | 0.637 | < 0.001^***^ |
|  |  |  |  |  |  |
|  |  |  |  |  |  |

**Figure S1.** Linear regression predicting green-lipped mussel shell weight from given shell length. Regression statistics summarised in the inset for n = 30 observations.


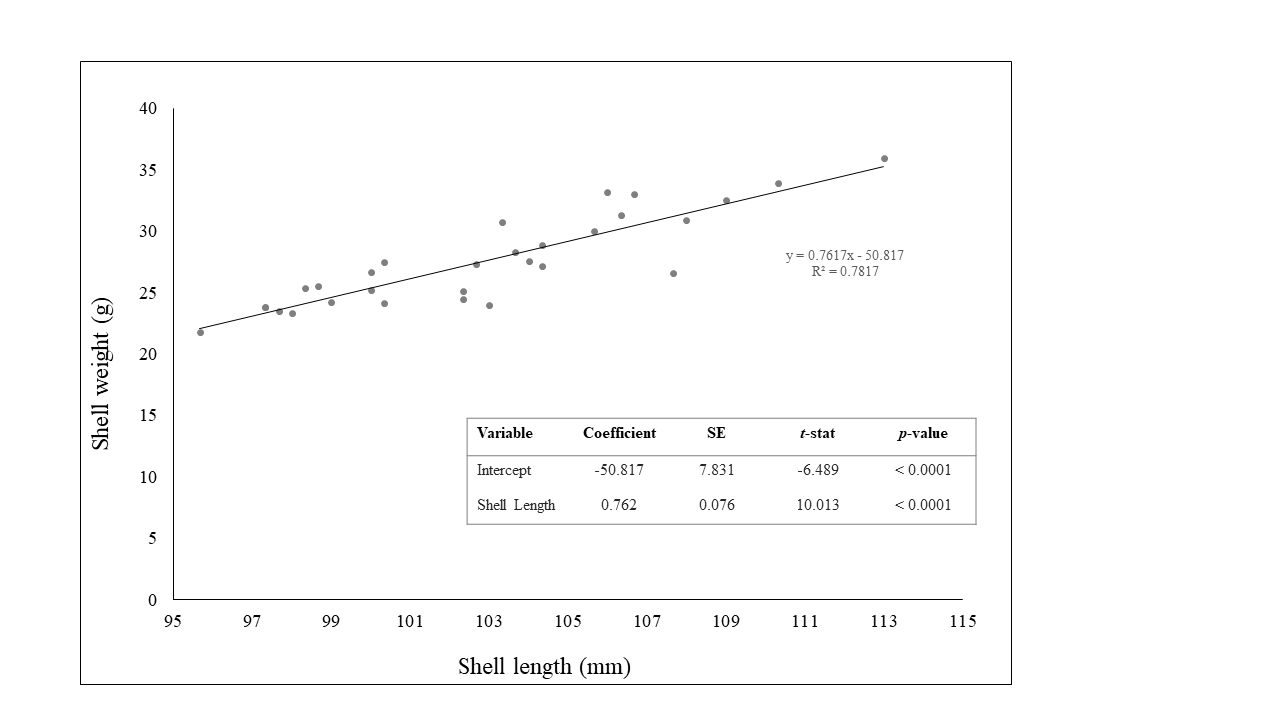


**Figure S2.** Results of 2-way ANOVA showing the effects of water quality (high vs. low suspended solids conc.) and location that mussels were collected from (site) on biodeposition rates. Analyses performed on untransformed data. Data represent the mean ± SE. Sites arranged over an increasing mud gradient, from outer bay to upper harbour. Site labels: Motuora = MR, Otarawao Bay = OT, Mahu Mid = MM, Pukapuka = PP, and Ngaio Bay = NB.


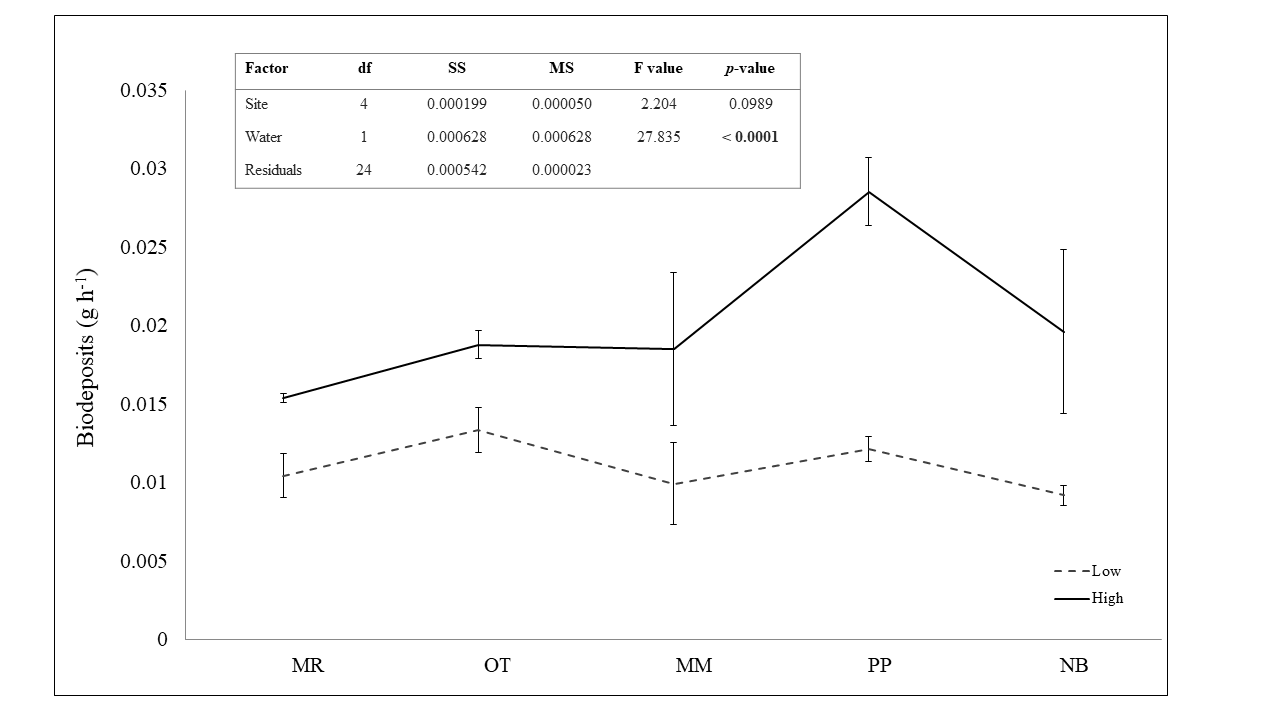


**Figure S3.** Linear regression plotting sediment oxygen demand vs. number of mussels in darkened, benthic flux chambers (0.25 x 0.25 m; volume = 41 L). Data obtained from Hillman et al., 2021 and Sea et al., 2021. A singular, adult green-lipped mussel is predicted to increase oxygen demand by 94.8 µmol O_2_ hr^-1^. Regression statistics summarised in the inset for n = 50 observations.


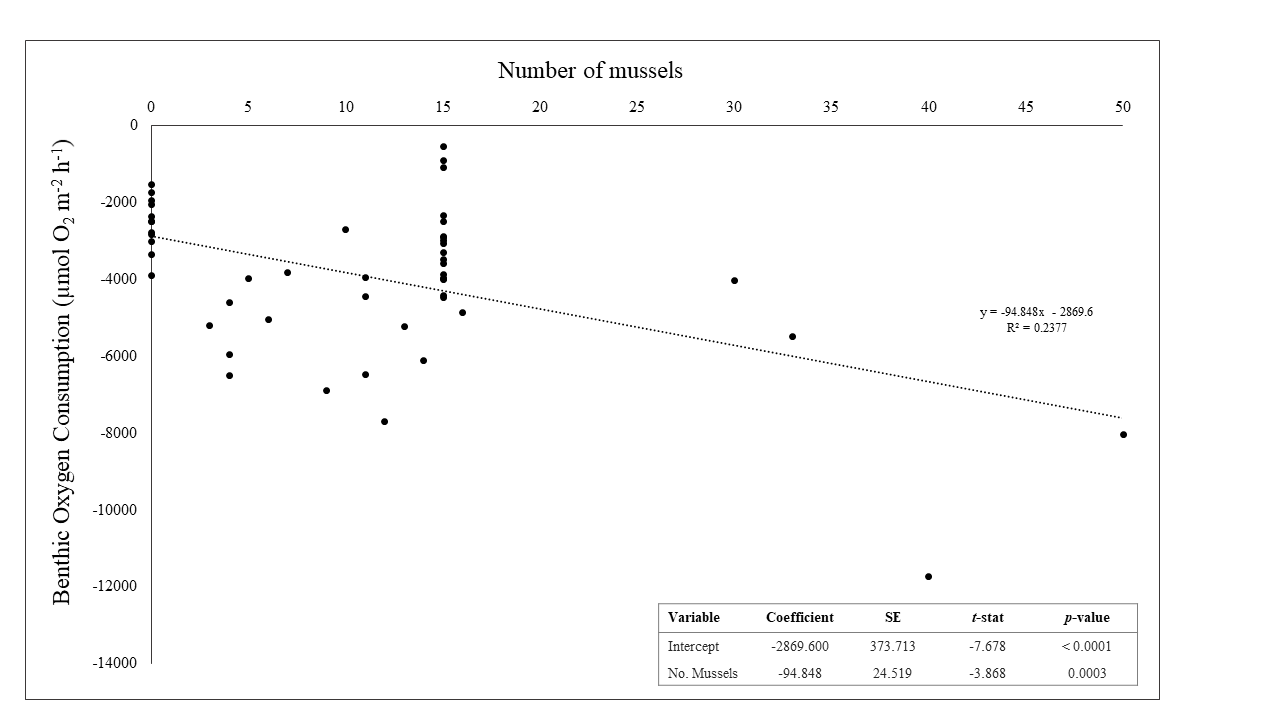

Supplement: Supplementary file 1 — Appendix S1 Supporting information [file GCB-28-5269-s001.docx]
